# Anterior Segment-Optical Coherence Tomography Bleb Morphology Comparison in Minimally Invasive Glaucoma Surgery: XEN Gel Stent vs. PreserFlo MicroShunt

**DOI:** 10.3390/diagnostics12051250

**Published:** 2022-05-17

**Authors:** Gloria Gambini, Matteo Mario Carlà, Federico Giannuzzi, Francesco Boselli, Giulia Grieco, Tomaso Caporossi, Umberto De Vico, Alfonso Savastano, Antonio Baldascino, Clara Rizzo, Raphael Kilian, Aldo Caporossi, Stanislao Rizzo

**Affiliations:** 1 Ophthalmology Unit, Fondazione Policlinico Universitario A. Gemelli, IRCCS, 00168 Rome, Italy; gambini.gloria@gmail.com (G.G.); giuliagrieco.md@gmail.com (G.G.); tomaso.caporossi@gmail.com (T.C.); umbertodevico@gmail.com (U.D.V.); alfonso.savastano@policlinicogemelli.it (A.S.); antonio.baldascino@policlinicogemelli.it (A.B.); aldocaporossi@yahoo.it (A.C.); stanislao.rizzo@gmail.com (S.R.); 2 Ophthalmology Unit, Catholic University “Sacro Cuore”, 00168 Rome, Italy; 3 Ophthalmology Unit, University of Verona, 37134 Verona, Italy; clararizzo2@gmail.com (C.R.); raphaelkilian8@yahoo.it (R.K.)

**Keywords:** glaucoma, micro-invasive glaucoma surgery, MicroShunt, mitomycin C, blebs, AS-OCT, InnFocus MicroShunt, PreserFlo MicroShunt, XEN Gel Stent, bleb morphology

## Abstract

Background: The purpose of this study is to compare the morphology of six-month follow-up blebs created by a subconjunctival glaucoma surgical device (XEN45) to those created by a PreserFlo MicroShunt with a sub-Tenon insertion, utilizing AS-OCT. Methods: A retrospective study of 29 eyes who underwent XEN45 implantation and 29 eyes who underwent PreserFlo MicroShunt implantation. The patients were analyzed at 24 h, 1 week, 1 month, 3 months and 6 months. At each visit, the maturation and morphological alterations of the blebs were observed, as well as connections with the IOP. Results: In both groups, IOP showed significant reduction at all follow ups (*p* < 0.0001). In XEN group, the most common bleb morphology in the immediate postoperative was the subconjuntival separation type (42%) followed by the uniform type (34%), with a trend inversion at 6 month follow up (51% of uniform type). On the contrary, the most common morphology after PreserFlo was the multiple internal layer (55%), which showed a tendency to reduce over time and was substituted by the microcystic multiform, whose percentage increased over time (17% at day 1 vs. 44% at month 6). Uniform appearance was associated by the posterior episcleral fluid (PEF) lake presence. Both horizontal and vertical diameters significantly increased over time. Conclusion: XEN and PreserFlo implantation resulted in the production of diffuse blebs with different characteristics, which may influence IOP lowering capacity and bleb revisions necessity over time.

## 1. Introduction

With the emergence of minimally invasive glaucoma surgery (MIGS) in recent years, surgical options for the treatment of glaucoma have expanded. Compared to standard glaucoma filtering surgery, these operations attempt to lower intraocular pressure (IOP) in a safer and less stressful way, reducing both intra-operative and post-operative complications linked with trabeculectomy (TB) [1,2]. 

**Figure 1:**
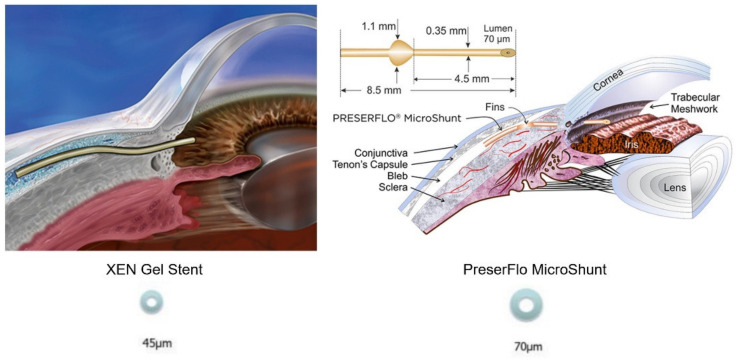
Graphical representation of XEN Gel Stent (**left**) and PreserFlo MicroShunt (**right**).

MIGS have different mechanisms of action. The PreserFlo MicroShunt (Santen, Osaka, Japan) (MicroShunt) [3,4] and the XEN 45 Gel Stent (Allergan Inc., Dublin, Ireland) (Xen) [5] drain aqueous humour into the sub-Tenon’s and subconjunctival area, respectively, creating a bleb which appear similar to the gold standard TB’s one, but with distinctive characteristics [6]. The XEN is a 6-mm long, flexible tube with a 45-µm lumen constructed of cross-linked porcine gelatine, normally implanted using an ab interno method with an injector [5]. The 8.5-mm PreserFlo MicroShunt, on the other side, is made of the stable and flexible polymer ‘SIBS’ (poly[styrene-blockisobutylene-block-styrene]), which has previously been utilized in the body for long-term implantation in cardiac stents, thanks to its biocompatibility [3]. It has a 70-µm lumen diameter and is implanted by an ab externo technique [4]. (Figure 1) The aim of this device is to keep a steady aqueous outflow via the tube’s lumen to a posterior point at least 7 mm distant from the limbus beneath Tenon’s capsule, without the use of a scleral flap or sutures to control the volume and direction of the aqueous flow, with several recent reports gathering its effectiveness in IOP control [7]. The Hagen–Poiseuille equation, stating that longe and thinner tubes will provide more resistance to flow than a shorter and wider ones, is the physical principle at the basis of both tubes’ structure, in order to reduce early postoperative hypotony for a given flow rate [8]. Moreover, both treatments are supplemented by intraoperative application or injection of mitomycin C (MMC) to lessen the risk of fibrosis.

Because the outflow volume of aqueous humor through the bleb determines the intraocular pressure (IOP)-lowering impact of a filtering procedure, bleb control is critical for surgical success. As it happened for trabeculectomy, a fibrotic reaction at the wound site in the subconjunctival tissue is the most significant risk factor for bleb failure [9]. Although current clinical classification systems (Picht & Grehn 1998; Indiana bleb Appearance Grading Scale [10], Moorfields Bleb Grading System [11]) are still useful for determining bleb height, extension, and vascularization, anterior-segment optical coherence tomography (AS-OCT) introduction allowed the assessment of the ultrastructure and differentiation of the type of bleb. Many studies have described fibrosis and scarring as the presence of high reflectivity areas within the bleb after trabeculectomy, as shown by AS-OCT images [12,13,14,15,16,17]. In recent years, several studies reported specific AS-OCT characteristics of the blebs formed after MIGS implantation, such as the absence of significant subepithelial fibrosis, epithelial thickening and flatter blebs [18], as well as different rates of multiple internal layer, microcystic multiform and uniform bleb morphology [6,19]. The exact ultrastructural properties of the blebs formed following PreserFlo and XEN implantation, as well as probable relationships between bleb morphology and IOP, still ignite the debate among glaucoma specialists.

The aim of our research is to outline the morphology of the blebs resulting from the implantation of a subconjunctival glaucoma surgery device (XEN45) with those resulting from PreserFlo MicroShunt, which features a sub-Tenon placement, using AS-OCT, in a six-months follow up. We indeed focused on ultrastructural changes in the bleb and correlated them to IOP variations and surgical success rates.

## 2. Materials and Methods

In this retrospective study, we included patients with primary open-angle glaucoma who underwent XEN45 Gel Stent or MicroShunt PreserFlo implantation at Fondazione Policlinico Universitario A. Gemelli-Rome between 27 January 2020 and 8 January 2021. All surgeries were perfomed by three experienced surgeons (S.R., A.C. and T.C.).

The presence of an open iridocorneal angle, evidence of glaucomatous optic nerve injury, and visual field (VF) deficits were all used to characterize primary open angle glaucoma (POAG). The research comprised individuals POAG who were on the most tolerated medication treatment and had increasing vision loss. The following were the inclusion criteria: best-corrected visual acuity (BCVA) of 20/200 or better; uncontrolled glaucoma on maximum tolerated medication with an IOP of 12 to 45 mmHg; phakic or pseudophakic patients treated with intracapsular lens implantation; and individuals who showed rapid and significant loss of visual function [mean deviation (MD), pattern standard deviation (PSD), visual function index (VFI), and glaucoma progression analysis (GPA) with the Humphrey Field Analyzer (HFA)]. In case of bilateral surgical indication, both eyes might be included if required, with at least one month between treatments.

Prior filtering operations (trabeculectomy), angle closure, pigmentary, pseudoexfoliative, uveitic, or neovascular glaucoma were all excluded from the study. Moreover, we considered the following exclusion criteria: age under 18, history of intraocular inflammation or any retinal abnormalities, history of non-glaucomatous optic neuropathy, optic nerve drusen, low-quality image due to hyper-mature cataract and unstable fixation. In case of concomitant cataracts, indications were given to perform XEN or PreserFlo implantation associated with phacoemulsification and IOL implantation. This study was conformed to the Declaration of Helsinki and approved by the Ethics Committee of Università Cattolica del Sacro Cuore. Informed written consent was obtained from all subjects. We included a total of 29 eyes who underwent XEN45 implantation and 29 eyes who underwent PreserFlo MicroShunt implantation, with the choice between the two to rely on surgeons’ preferences or devices’ availability

All patients’ demographics (age, sex, surgery date, laterality, and diagnosis), type of surgery (combined or stand-alone), and ocular parameters, were collected at the preoperative visit, including BCVA assessed with the Snellen chart, IOP at which the decision for surgery was made (measured with Goldmann applanation tonometer) and number of glaucoma medications at the preoperative visit. The post-operative IOP, BCVA, and AS-OCT measurements, as well as the size of the bleb (described in detail in the AS-OCT section), were analyzed throughout follow-up (24 h, 1 week, 1 month, 3 months and 6 months). The number of patients with hypotony, those who needed glaucoma medication, and the total amount of drugs were also gathered.

IOP > 21 mmHg or a <20% drop below preoperative baseline on 2 consecutive follow-up visits after 3 months, or hypotony < 5 mmHg on 2 consecutive follow-up visits after 3 months, loss of light perception vision or glaucoma reoperation, were all considered surgical failures. Reoperation for glaucoma was defined as further glaucoma surgery, including slit lamp needling operations. Cases that required an additional glaucoma intervention were only included in the postoperative analysis up to the moment of the decision to intervene.

Surgical success was defined as a postoperative IOP ≤ 18 mmHg at 2 consecutive follow-ups after 3 months. A complete success was defined for eyes which were not undergoing further medical treatment, while a qualified success was defined as eyes that had not failed but needed medical treatment to manage IOP.

### 2.1. Surgical Techniques

An ab interno approach was used for XEN implantation. A 0.2 mg/mL Mitomycin C (MMC) solution was injected in the nasal superior quadrant, beneath Tenon’s capsule, rubbed over the expected insertion location and thenwashed with balanced salt solution (BSS). In the inferotemporal quadrant, a primary 1.2-mm corneal paracentesis incision was created, and the anterior chamber (AC) was filled with an Ophthalmic viscosurgical device (ProVisc, Alcon Laboratories Inc., Fort Worth, TX, USA) (OVD). The injector was then guided through the incision across the AC towards the superonasal quadrant, and implanted through the sclera in the subconjunctival area, with 1–2 mm of tube still visible in the AC. Finally, a goniolens was used to check placement shortly after implantation.

PreserFlo implantation was conducted through an ab-externo procedure. In the superonasal or superotemporal quadrant, a fornix-based conjunctival flap was created. After forming a deep sub-Tenon’s pocket, 0.2 mg/mL MMC was administered to the scleral surface for 3 min using numerous MMC-soaked Lasik shields positioned into the pocket. The region was carefully washed BSS after the shields were removed. After marking 3 mm from the limbus, a 1-mm triangular knife was used to make a scleral pocket, following which a 25-gauge needle was inserted into the pocket to construct a needle track into the AC. The Micro-Shunt was put into the AC through the needle track, with the device’s fin tucked snugly into the pocket. Tenon’s capsule and conjunctiva were sutured in a watertight manner after flow was checked.

### 2.2. AS-OCT Protocol

At 24 h, 1 week, 1 month, 3 months and 6 months follow-up visits after surgery, an independent examiner collected AS-OCT bleb scans (Solix full-range OCT, Optovue Inc., Freemont, CA, USA). Nonetheless, due to the retrospective nature of the study, not all patients got scans at each time point. The AS-18 mm OCT’s radial scan feature was used to capture pictures centered on the bleb area. To disclose the filtering bleb, the patient was advised to gaze down while the upper eyelid was pushed up. The same examiner took all of the photos without putting any extra strain on the eye. Finally, we selected two images based on the degree of bleb visible and the lack of lid and motion artifacts. The horizontal axis (parallel to and 3 to 5 mm distant from the limbus) and the vertical axis (perpendicular to the limbus) were used to measure the blebs. The maximum bleb dimensions were calculated by averaging the horizontal and vertical measurements using the OCT system’s caliper tool. Microcysts, layers of aqueous flow, and/or a posterior aqueous lake observed along the horizontal axis were used to determine the bleb’s horizontal extent. The whole set of measurements was completed by two distinct observers (G.G. and M.M.C.) and the final data was produced by averaging their findings. When the bleb width surpassed the AS-OCT display for bleb measurements, the horizontal measure was taken from one side to the other of the picture, even if the boundaries could not be determined.

The two observers separately judged the degree of internal reflection. The blebs were further examined based on their morphological properties, following Nakano et al. classification for trabeculectomy blebs, and were divided into uniform and multiform blebs [20]. Uniform pattern was characterized by less presence of microcysts or multiple layers of fluid across the bleb wall, with the non-reflective area confined into a single, larger space. On the other side, areas of hyporeflectivity in AS-OCT images of blebs with multiform walls were classified as one of three structural features of the developing bleb wall, focusing on the predominating feature: multiple internal layer, microcystic multiform and subconjunctival separation from the conjunctival epithelium. A review of all bleb morphologies is available in Table 1.

### 2.3. Statistical Analysis

The sample size of each considered group was evaluated using the G-Power software package (Version 3.1.9.6). Assuming a minimum difference of 15%, a residual standard deviation of 10%, a power of 0.08 and an alpha of 0.05 to highlight the differences, the required smallest population size was 18 patients for each group. The statistical analysis was performed using GraphPad PRISM Software (Version 9.0; GraphPad, La Jolla, CA, USA). Shapiro-Wilk test was used to assess the normality of our sample, with *p* > 0.05 set to verify the null hypothesis. We conducted an Analysis of Variance (ANOVA) and employed the Dunnett’s multiple comparison test with the Geisser-greenhouse correction for matched pairs. A Tukey test, computing confidence intervals, was used to compare the difference between each pair of means, in non-matched pairs. Chi-square and Fisher’s exact test were used for contingency analysis. Furthermore, correlation and regression analyses were conducted for continuous variables. Quantitative values were expressed as mean ± SD and a *p* value < 0.05 was considered statistically significant. A designated confidence interval (CI) of 95% was used.

## 3. Results

A total of 58 eyes were included in the study, half in the XEN group, half in the PreserFlo group. The study population’s mean age at the time of surgery was 72.7 ± 5.6 years; laterality (RE/LE) was 31/27; sex (M/F) was 28/30. Mean preoperative IOP treated with anti-glaucoma drugs was 22.1 *±* 3.1 (range, 16–29 mmHg, with no statistical differences among XEN and PreserFlo groups, *p* > 0.99) on a mean number of topical drugs of 2.6 *±* 0.9; mean BCVA was 0.57 *±* 0.3; and mean CCT was 538 *±* 42 µm. A total of 41 patients (71%) were already pseudophakic. 10 patients (17%) underwent combined surgery. Demographic charachteristics are visible in Table 2.

### 3.1. XEN Gel Stent Results

Mean IOP in the XEN group dropped from 22.1 *±* 2.9 mmHg (range 17–29) at baseline to 10.6 *±* 2.7 mmHg at day 1, 12.5 *±* 2.6 mmHg at week 1, 14.2 *±* 2.1 mmHg at 1-month follow up, 13.8 *±* 2.0 at 3-month follow up and 14.2 *±* 2.0 at 6-month follow up. At all time intervals, the difference from baseline was significant (mean differences of 11.7 mmHg at 24 h, 9.6 mmHg at 1 week, 7.9 mmHg at 1 month, 8.3 mmHg at 3 months and 7.9 at 6 months; *p* < 0.0001 at all follow ups). Pairwise analyses of the IOP by age (under 75 years and over 75 years) or sex revealed no significant differences (*p* = 0.68; *p* = 0.42). Moreover, stand-alone procedure compared with combined phacoemulsification revealed no statistical differences regarding IOP decrease (*p* = 0.24).

Complete surgical success rates were 51% and 44% at the 3- and 6-month follow ups, respectively. The probability of qualified success was 78% after 3 months and 73% after 6 months.

### 3.2. PreserFlo MicroShunt Results

PreserFlo MicroShunt implantation reduced IOP from a mean baseline of 22.0 (range 16–28) mmHg to 8.1 *±* 2.8 mmHg after 24 h, 8.7 *±* 2.0 mmHg after one week, 11.3 *±* 2.1 mmHg after one month, 12.1 *±* 2.2 after three months and 12.9 *±* 2.1 at the 6-month follow up. At all time intervals, the difference from baseline was substantial (mean differences of 13.9 mmHg at 24 h, 13.31 mmHg at 1 week, 10.7 mmHg at 1 month, 9.9 mmHg at 3 months and 9.1 at 6 months, *p* < 0.0001 at all follow ups). Similar to XEN group, pairwise analyses of the IOP by age or sex didn’t show any statistical differences (*p* = 0.58 and *p* = 0.47, respectively), as well as possible combined cataract surgery (*p* = 0.17).

We found lower IOP values in the MicroShunt group at all time-points; however, this difference was only statistically significant at day 1, week 1 and month 1 follow ups (*p*= 0.0087, *p* = 0.0001, *p* = 0.0005, respectively).

Complete surgical success rates in this group were 62% and 56% at the 3- and 6-month follow ups, respectively. The probability of qualified success was 82% after 3 months and 79% after 6 months.

**Figure 2:**
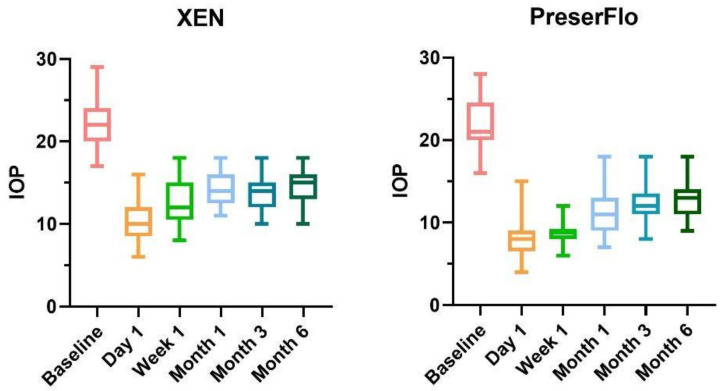
Box and plot diaphgrams of IOP variations in the two subgrops, with indication of every follow up. IOP = intraocular pressure.

Figure 2 shows IOP variations among different follow ups in the two groups.

### 3.3. Medication

In the XEN and PreserFlo groups, the mean number of IOP-lowering drugs decreased from 2.5 *±* 1.0 and 2.7 *±* 0.8 at baseline to 0.7 *±* 1.1 and 0.4 *±* 1.2 at 6 months, respectively. At the end of the study, 44 percent (13/29) of patients in the XEN group and 56 percent (16/29) of patients in the PreserFlo group were free of IOP lowering medication (*p* = 0.14). At the 6-month follow-up, none of the patients in either group had utilized oral acetazolamide.

### 3.4. AS-OCT Measurements

Table 3 shows the AS-OCT morphological categories of XEN and PreserFlo blebs.

In XEN group, the most common bleb morphology in the immediate postoperative was the subconjuntival separation type (42%) followed by the uniform type (34%). Nevertheless, the microcystic multiform and multiple internal layer morphology were present in our case (14% and 10%, respectively). The subconjuntival separation, during the follow up, presented a downward trend (24% at 6 month), while the uniform type presented an upward trend (51% at 6 month), becoming the most prevalent at the last follow-up.

**Figure 3:**
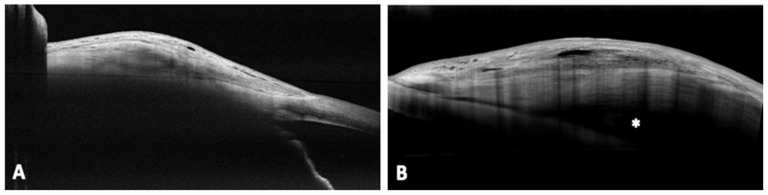
AS-OCT image of a uniform bleb after 6 month from XEN implantation (**A**), and uniform morphology of a PreserFlo bleb with an associated PEF lake, indicated by the white asterisk, after 6 months (**B**). AS-OCT = anterior segment-optical cohernce tomography; PEF = posterior episcleral fluid.

On the contrary, the most common morphology after PreserFlo procedure, at day 1, was the multiple internal layer (55%), which showed a tendency to reduce over time until it settled down around one third (37%), stable from month 1 onwards. At the last follow up PreserFlo’s most representative bleb was the microcystic multiform, whose percentage increased over time (17% at day 1 vs. 44% at month 6). The uniform pattern was well represented in our case series (7% at day 1 vs. 21% at month 6). Furthermore, all functional blebs in uniform subgroup featured the presence of an episcleral cavity, extending posterior to the limbus and seen as a nonreflective area between the connective tissue of the bleb wall and sclera. We denoted this cavity as the posterior episcleral fluid (PEF) lake. Example of bleb’s morphologies are visible in Figure 3.

In both groups the blebs’ size was evaluated and showed a difference between XEN and PreserFlo morphologies. Regarding the horizontal and vertical diameters of the bleb, the Shapiro–Wilk test showed that the data followed a normal distribution. In the first group, a more flat and diffuse bleb was the main representation, with the mean horizontal bleb diameter at 6 month of 3514 ± 861 µm, and a vertical diameter of 359 ± 140 µm. PreserFlo MicroShunt implantation instead determined the formation of defined blebs (mean horizontal bleb diameter at 6 month 3012 ± 560 µm), with greater thickness diameter (mean vertical diameter 432 ± 124 µm).

A report of all horizontal and vertical blebs’ diameters at every follow up is showed in Table 4. In the XEN group, horizontal diameter showed an increase over time compared to baseline (mean differences of 340, 529, 1209 and 1546 µm at 1 week, 1 month, 3 month and 6 month follow ups), which appeared statistically significant at month 1, month 3 and month 6 follow ups (*p* = 0001, *p* < 0.0001 and *p* < 0.0001, respectively), but not at 1 week (*p* = 0.10). Similarly, vertical diameters increased during follow ups (mean differences of 38, 98, 150 and 191 µm) appearing significant at every follow up (*p* = 0.001 at week 1, *p* < 0.0001 at every other follow up).

In the PreserFlo group, bleb horizontal diameter increased in a non significant way from day 1 to week 1 (mean difference 160 µm, *p* = 0.48), but then significantly at month 1 (mean difference 588 µm, *p* < 0.0001), month 3 (mean difference 856 µm, *p* < 0.0001) and month 6 (mean difference 1298 µm, *p* < 0.0001). Vertical diameter of the bleb, on the other hand, underwent a significant increase at every follow up (mean differences of 79, 140, 199 and 224 µm, *p* < 0.0001 for all of them).

Horizontal diameters were characterized by much more variability over time, and showed a significant difference among the two groups at last follow up, with XEN group showing higher values (mean difference 412 µm, *p* = 0.03). The vertical bleb diameters were less variable, increasing gradually from day 1 to month 6. The comparison among the two groups showed a tendency of higher vertical diameters in the PreserFlo group, although not statistically significant (mean difference of 72 µm, *p* = 0.14).

At last, we analyzed the correlation between the IOP and the size of the bleb formed following XEN and PreserFlo implantation. A negative but non-significant correlation was discovered between the horizontal bleb diameter and the IOP in the XEN group from baseline to month 6 (r = −0.18, *p* = 0.10). In the PreserFlo group, a weak and negative correlation was found between the vertical diameter and the IOP when comparing baseline to month 6 follow up, even if not statistically significant (r = −0.24, *p* = 0.38).

### 3.5. Post-Operative Interventions and Complications

In both groups, early self-limiting hyphema was often recorded (rates of 20 and 24% of eyes, respectively). The rate of hypotony at day 1 and week 1 was found in 6 (20%) and 4 (14%) of cases in the XEN group, respectively, and in 8 (28%) and 4 (14%) of eyes in the PreserFlo group, respectively (*p* = 0.28). Hypotony had resolved in all instances at the 1-month follow up. Two patients in the XEN group and one patient in the PreserFlo group required AC reformation in the first postoperative week, while one incidence of choroidal effusion was detected in both groups, being successfully managed with conservative therapy.

Bleb needling was performed on 6 eyes (21%) in the Xen group and 2 eyes (7%) in the PreserFlo group, respectively (*p* = 0.14). Two eyes (7%) in the XEN group required bleb revision, compared with one eye (3%) in the PreserFlo group. One instance in the XEN group needed surgical stent placement revision, due to migration towards the path of the AC. In the MicroShunt group, there were no incidents of device exposure or migration.

## 4. Discussion

Surely, the features of the bleb are of extraordinary importance for understanding the clinical functionality of MIGS. The aim of our study was to understand the correlation between the morphology of the bleb and the functionality of XEN and PreserFlo devices in early and medium follow ups. We studied the morphology of the bleb through a non-invasive and repeatable technique such AS-OCT, which demonstrated its effectivenessin the evaluation and study of blebs’ morphology in various glaucoma surgery techniques.

In the XEN group, multiform bleb morphology, with a prevalence of subconjunctival separation pattern, was the most common in the early follow ups, but gave way to the uniform patter which became the commonest (51%) at the 6 month follow up. These results are similar to that described by Lenzhofer et al., who reported a rate of 48% uniform blebs already after 2 weeks after XEN implantation [6]. Our results suggest that in AS-OCT, bleb morphology with subconjunctival separation is linked with lower mean IOPs in the early postoperative period, identifying the OCT feature of a well-functioning early bleb. Similarly to Lenzhofer et al. reports, small cysts with diffuse fluid distribution seem to indicate less fibrosis and, as a result, a reduced bleb outflow resistance (lenzhofer).

On the other side, at all time points a multiform bleb morphology was the most common AS-OCT pattern in the PreserFlo group, with the presence of multilayered, loosened stroma being more common in the very early postoperative period than later in follow-up (55 vs. 31%), likely due to the fibrotic response induced by surgical trauma. However, bleb maturation during the study period determined the formation of fluid gaps and subepithelial microcysts, which became the most common form of the bleb at the end of the study (increased from 17 to 44%). The low reported percentage of uniformity compared to TB and XEN implantation was not confirmed in our study, in which uniform pattern was well represented at last follow up (24%). This evidence is in contrast to the evidence of Barbera et al. in which the presence of a uniform bleb was infrequent with an observational rate of 3.5% after the first week, but did not increase over time [21]. Interestingly, in our study uniform bleb morphology was linked with an AH drainage route surrounding the tube, beneath Tenon’s capsule, which determined the formation of the posterior episcleral fluid (PEF) lake. This aqueous reservoir, resembling the episcleral cavity reported in successful trabeculectomy blebs [22], was indeed index of bleb functionality even after PreserFlo implantation. On the other side, this appearence was not reported in XEN’s blebs in our study, which was consistent with several researches reporting results after ab interno method for XEN device [23,24]. A recent research conducted by Dangda et al. showed that a sub-Tenon placement of the XEN45 might result in PEF lake formation in all cases of uniform blebs patterns [25]. We hyphotesize that the high incidence of full surgical success with big PEF lake development might depend on to the extensive intra-operative sub- Tenon’s dissection as well as the MMC action on sub-Tenon’s tissues, which let the AH to flow towards a single reservoir.

In our research, bleb diameters increased from the very early postoperative phase to the last follow up, in both groups. In XEN’s eyes, the horizontal bleb diameter expanded more than PreserFlo’s ones, which confirms different bleb appearences reported in literature [6,21,24]. In comparison to patients who did not achieve successful IOP control, AS-OCT studies on blebs following XEN surgery found that the maximum height of the bleb and the total area of cystic hyporeflective spaces were both greater, while bleb internal reflectivity was lower in patients with successful IOP control [26,27]. In our study, the AS-OCT measurements of the bleb’s horizontal and vertical diameters did not reveal a significant link with the reduction in IOP in both XEN and PreserFlo groups suggesting that not only is the size of the bleb surrounding the tube most likely to induce IOP drop, but the bleb morphology.

Previous studies on TB’s blebs found a link between smaller blebs and greater IOP levels following TB using computational fluid flow modeling [28]. In this model, the fluid must travel away from the principal bleb before it can be entirely absorbed in smaller TB blebs, but the fluid movement is restricted by a reduced hydraulic conductivity, which causes a rise in IOP. Larger blebs were anticipated to cause lower IOP levels in the same model, but with a high risk of hypotony in TB blebs due to the absence of defining cutoff values. According to Gardiner’s model created for TB, the predicted incidence of hypotony following PreserFlo implantation should be greater, based on the bleb shape described in majority of patients. The low incidence of hypotony despite the high frequency of big diffuse blebs might be explained by the PreserFlo’s steady flow rate, the resistance to flow through the implant’s lumen, which differs from TB and other devices like the XEN 45 [21]. In these devices, the flow rate is primarily determined by the lumen diameter and tube length, according to the Hagen–Poiseuille equation. Based on these findings, the resistance to flow through the PreserFlo is predicted to be 2 mmHg/L/min, in contrast to the 8.5 mmHg/L/min for the XEN 45 [29]. These two devices are not only different in terms of flow resistance, but also in terms of surgical technique. According to Lee et al., the classical ab interno approach utilized with the XEN implant resulted in considerably higher outflow resistance as compared to ab externo implantation of the identical device (0.8 ± 0.11 mmHg/L/min vs. 0.42 ± 0.05 mmHg/L/min) [30]. The higher outflow resistance associated with the ab externo technique used for the XEN implant, especially when it ends up being implanted intraconjunctivally, as well as the higher resistance to flow through the tube, may explain the uniform bleb rate of 48 percent reported by Lenzhofer et al. [6,19], as well as the results reported by Teus et al. [18], which included significantly flatter XEN blebs. (teus et al.) Further analysis on the ab interno approach for the XEN implant, showed better IOP control with deeper insertion (intra–sub–Tenon) than with superficial placement (intraconjunctival).

Despite the fact that both the XEN and PreserFlo implants are tubes with a continuous fluid flow, our results are similar to those reported in other researches, implying that the blebs that grew after PreserFlo implantation resemble those that grow after TB more than those that occur after XEN implantation [21]. Most likely, the PreserFlo’s lower resistance to flow compared to the XEN, as well as the surgical ab externo technique, which reduces outflow resistance and allows the surgeon more control over positioning of the tube beneath Tenon’s capsule at a posterior location (approximately 7 mm from limbus), produce intermediate aqueous humour drainage at a low pressure, which maintains over time (12.9 *±* 2.1 at the 6-month follow up). According to Gardiner [28], scar development at low pressures begins above the bleb, where the pressure is greater, but not on the horizontal margins of the bleb, where the pressure is lower: with a low pressure over time, the PreserFlo’s bleb may slowly and gradually increase its horizontal bounds, reducing resistances to AH flows. Given these findings, it is reasonable to speculate that the standardized ab externo technique used for PreserFlo implantation, in addition to the deeper and posterior localization of the distal end of the tube and its lower resistance to flow compared to the XE, may be related to the higher number of multiform blebs and lower rate of surgical revision reported in literature and seen in this research. Moreover, repeatable morphology in the majority of Preserflo’s bleb similar to those described for functioning TB blebs [20].

The increased incidence of success following the creation of filtering blebs in MIGS devices (qualified success of 79% and 73% in PreserFlo and XEN groups in our research, respectively) might be related to a reduction in the inflammatory response after those minimal surgical procedures. Inflammation, in fact, is a major contributor to glaucoma surgical failure [31]. Another aspect that seems to reduce the inflammatory response in the instance of the PreserFlo is the SIBS polymer, which was originally used for coating cardiac stents (TAXUS stent), aiding for reduced scarring and chronic inflammation, with no biodegradation and little tissue reactivity [32]. In the early postoperative phase, the existence of multiform, broad blebs with low IOPs, in conjunction with a polymer that generates little inflammation and MMC to modulate the fibrotic response, might be a predictor of long-term success [21].

At the end, both procedures were found to be effective in IOP lowering and reducing the number of postoperative medications. Our results suggest that XEN and PreserFlo implants may have similar IOP-lowering potential, with a tendency of lower IOP values in the PreserFlo groups, and lower cases of postoperative needlings. Moreover, while postoperarive hypotony was prevalent in both groups (20 and 28% at day one, respectively), it resolved in all instances at the 1-month follow up.

As far as we know, this is the first research to analyze the ultrastructural AS-OCT difference among two of the most frequent MIGS devices. Surely this study has some limitations, starting from its retrospective design and its small case series. Moreover, bleb morphology evaluation may still have been influenced by subjective backgrounds of the operators, due to the lack of objective algorythms of calculation. At last, horizontal and vertical diameters may not be able to perfectly define the shape of a bleb, thus still resulting an indicative parameter.

## 5. Conclusions

In conclusions, both XEN and PreserFlo implantation for glaucoma resulted in the production of diffuse blebs with different characteristics. Subconjunctival separation appearance was typical in the early XEN’s blebs, and generally maturated towards a uniform morphology at the 6 month follow ups. PreserFlo’s blebs, on the other side, were situated beneath Tenon’s capsule and presented a multiform shape on AS-OCT in the majority of patients, as already described in literature. Moreover, PreserFlo’s bleb maturation showed increased mycrocistic subepithelial appearance and demonstrated a much more repeatable morphology, in the majority of cases similar to those described in well functioning TB blebs. Surprisingly, our study showed a well represented uniform morphology in the PreserFlo group, which represented a good prognostical factor, when associated to the presence of the PEF lake.

Finally, we speculate that, based on our findings, an extensive analysis of blebs’ morphologies in these MIGS may become an important prognostic factor to identify which conditions are more likely to fail over time, thus offering the possibility for a prompt re-treatment. Further studies are surely needed to assess how the bleb maturation process may continue in a long-term follow-up.

## Figures and Tables

**Table 1:** Bleb morphologies and their typical appearances.

Bleb Morphology	Characteristics
Uniform	No fluid-filled hyporeflective spaces in subconjunctival space.
Multiple internal layer	Hyporeflective spaces in deep and superficial layers of conjunctiva with channel of fluid parallel to the surface of the sclera.
Mycrocystic multiform	Multiple cystic hyporeflective areas in deep layer separated by this septae.
Subconjunctival separation	Multiple small hyporeflective spaces in superficial conjunctival space.

**Table 2:** Baseline demographic and clinical characteristics of the study population.

	XEN (*n* = 29)	PreserFlo (*n* = 29)	*p*
Study eye, OD, no (%)	16 (55%)	15 (52%)	0.86
Mean age, yrs	73.2 ± 4.8	72.2 ± 5.7	0.68
Gender, male, no (%)	13 (45%)	15 (52%)	0.47
BCVA ± SD, decimals	0.61 ± 0.3	0.54 ± 0.3	0.32
Mean IOP ± SD; mmHg	22.1 ± 2.9	22.0 ± 3.3	0.81
Mean CCT ± SD, µm	535 ± 51	541 ± 32	0.79
Mean no. of IOP-lowering drugs ± SD	2.5 ± 1.0	2.7± 0.8	0.53
History of phacoemulsification (%)	19 (66%)	22 (76%)	0.24
History of SLT (%)	6 (21%)	7 (24%)	0.36

BCVA = Best Corrected Visual Acuity; IOP = intraocular pressure; CCT = central corneal thickness; SLT = selective laser trabeculoplasty.

**Table 3:** Bleb morphology classification of the XEN and PreserFlo blebs at day 1, week 1, month 1, month 3 and month 6 follow ups.

	XEN					PreserFlo			
	Uniform	Microcystic Multiform	Subconjuntival Separation	Multiple Internal Layer		Uniform	Microcystic Multiform	Subconjuntival Separation	Multiple Internal Layer
Day 1 (*n* = 29)	10 (34%)	4 (14%)	12 (42%)	3 (10%)	Day 1 (*n* = 29)	2 (7%)	5 (17%)	6 (21%)	16 (55%)
Week 1 (*n* = 29)	9 (31%)	3 (10%)	11 (38%)	6 (21%)	Week 1 (*n* = 28)	3 (10%)	5 (18%)	5 (18%)	15 (54%)
Month 1 (*n* = 26)	13 (50%)	2 (8%)	8 (31%)	3 (11%)	Month 1 (*n* = 27)	4 (15%)	9 (33%)	4 (15%)	10 (37%)
Month 3 (*n* = 28)	14 (50%)	2 (7%)	8 (29%)	4 (14%)	Month 3 (*n* = 28)	5 (18%)	12 (43%)	2 (7%)	9 (32%)
Month 6 (*n* = 29)	15 (51%)	3 (10%)	7 (25%)	4 (14%)	Month 6 (*n* = 29)	6 (21%)	13 (44%)	1 (4%)	9 (31%)

**Table 4:** Horizontal and vertical bleb diameters after XEN and PreserFlo implantation at day 1, week 1, month 1, month 3 and month 6 follow ups.

	XEN (*n* = 29)				PreserFlo (*n* = 29)			
	Horizontal Diameter (µm ± SD)	*p*	Vertical Diameter (µm ± SD)	*p*	Horizontal Diameter (µm ± SD)	*p*	Vertical Diameter (µm ± SD)	*p*
Day 1	1967 ± 775		168 ± 136		1804 ± 675		208 ± 115	
Week 1	2307 ± 673	0.10	205 ± 121	0.001	1964 ± 545	0.48	287 ± 117	<0.0001
Month 1	2496 ± 580	<0.0001	266 ± 134	<0.0001	2392 ± 632	<0.0001	348 ± 137	<0.0001
Month 3	3176 ± 615	<0.0001	318 ± 140	<0.0001	2660 ± 672	<0.0001	407 ± 126	<0.0001
Month 6	3514 ± 861	<0.0001	359 ± 125	<0.0001	3102 ± 760	<0.0001	432 ± 143	<0.0001

## Data Availability

The data that support the findings of this study are available from the corresponding author, MMC, upon reasonable request.
